# Severe fatigue due to valproate-induced hypothyroidism in a case of bipolar disorder

**DOI:** 10.1186/s12991-020-00299-y

**Published:** 2020-09-05

**Authors:** Tadashi Kanamori, Masahiro Suzuki, Yoshiyuki Kaneko, Kouju Yamada, Hideyuki Kubo, Makoto Uchiyama

**Affiliations:** 1 Department of Psychiatry, Nihon University School of Medicine, 30-1 Oyaguchi-Kamicho, Itabashi-ku, Tokyo, 173-8610 Japan; 2 Kunpukai Yamada Hospital, Tokyo, Japan; 3 Tokyoadachi Hospital, Tokyo, Japan

**Keywords:** Bipolar disorder, Hypothyroidism, Symptomatic, Valproate

## Abstract

**Background:**

Valproate-induced hypothyroidism is a rare condition and has been considered asymptomatic. Here, we report a case of bipolar I disorder who developed symptomatic valproate-induced hypothyroidism.

**Case presentation:**

A 44-year-old woman with bipolar I disorder complained of severe fatigue after starting valproate. She showed a hormonal pattern of central hypothyroidism. Thyroid autoantibodies were negative, and no pituitary abnormality was seen on magnetic resonance imaging. After stopping valproate, her severe fatigue rapidly improved with normalizing thyroid function.

**Conclusions:**

Our case suggests that valproate-induced hypothyroidism should be considered when patients complain of excessive fatigue under treatment with valproate.

## Background

Valproate, which is recognized to be much safer than lithium, the archetypal mood stabilizer, in terms of effects on thyroid function, is widely used in the treatment of bipolar disorder [1]. However, previous reports have suggested that valproate can also cause hypothyroidism [2, 3]. Here, we report a case of bipolar disorder showing severe fatigue due to valproate-induced hypothyroidism.

## Case presentation

A 44-year-old woman with bipolar disorder was referred to our outpatient clinic 2 months after her first manic episode. She developed bipolar disorder with depressive episodes when she was 42 years old. Her first depressive episode was ameliorated with 3 months’ treatment with sertraline. She had no previous episodes of hypomania or mixed states, and no history of thyroid disease. When she presented at our clinic, she had been treated with quetiapine for 1 month, but still had elevated mood, irritability, and mood-congruent delusions. According to the Diagnostic and Statistical Manual of Mental Disorders, 5th edition, we diagnosed her as having bipolar I disorder, and added 200 mg of valproate on 100 mg of quetiapine. Valproate was later increased to 400 mg, after which, her manic symptoms ameliorated.

Despite her mood being well controlled, she developed severe fatigue 30 days after starting valproate. She had no depression-related symptoms other than fatigue, and no findings suggesting sedation with valproate such as somnolence or impaired attention. Her blood concentration of valproate was 24.2 μg/mL, which was not at the toxic level (normal range 50–100 μg/mL). She had never had a similar reaction to previously used psychotropics. A physical examination and laboratory test found no abnormalities except for low values of free thyroxine 0.50 ng/dL (F-T4; normal range 0.8–1.5 ng/dL) and free triiodothyronine 1.85 pg/mL (F-T3; normal range 2.0–3.8 pg/mL). Although F-T4 was decreased, thyroid-stimulating hormone 2.97 μU/mL (TSH; normal range 0.34–3.8 μU/mL) was within the normal range, suggesting central hypothyroidism. Since other fatigue-causing medical conditions and medications were ruled out by systematic evaluations, we considered that the severe fatigue was associated with hypothyroidism. Thyroid autoantibodies were negative, and gadolinium-enhanced magnetic resonance imaging of the pituitary gland showed no evidence of a pituitary lesion. Considering that her F-T4 levels progressively decreased with increasing doses of valproate (F-T4 0.70 ng/dL under 200 mg and F-T4 0.50 ng/dL under 400 mg), we suspected that her hypothyroidism was caused by valproate. Therefore, we stopped valproate 33 days after its introduction. Her severe fatigue then improved, completely disappearing in about 20 days. A laboratory test 35 days after stopping valproate confirmed that her thyroid function had normalized (TSH 2.17 μU/mL, F-T3 2.99 pg/mL, F-T4 1.10 ng/dL). For the next 12 months, she had no recurrence of mood episodes, hypothyroidism, or fatigue under maintenance treatment with risperidone and carbamazepine.

## Discussion

In this case, severe fatigue was relieved by discontinuation of valproate with normalizing thyroid function. In addition, her severe fatigue and hypothyroidism were clearly exacerbated depending on the dose of valproate. Therefore, we determined that the use of valproate was associated with her hypothyroidism and severe fatigue. Furthermore, we assessed the causal relationship between the use of valproate and the observed reactions using the Adverse Drug Reaction Probability Scale developed in 1981 by Naranjo and coworkers (i.e., the Naranjo Scale) [4]. This scale has been widely used to assess causality for adverse drug reactions. The Naranjo Scale is composed of 10 questions that are answered as either “Yes”, “No”, or “Do not know”. The total score ranges from –4 to 13, and a reaction is considered definite when the score is ≥ 9, probable if 5–8, possible if 1–4, and doubtful if ≤ 0. In this case, the causality of the association between valproate and hypothyroidism was estimated as being “Probable” based on the total score of 8. We did not re-administer valproate or perform a placebo challenge; therefore, we answered “Do not know” for two of the 10 questions. Our case showed fatigue, but no other symptoms of hypothyroidism, such as weight gain or myxedema; this may have been due to the mild decline in thyroid hormone levels.

Valproate-induced hypothyroidism has been considered to be asymptomatic, even if it occurs [2, 3]. To our knowledge, this is the second report of a case of symptomatic valproate-induced hypothyroidism. Similar to our case, Rao et al. reported a case of bipolar disorder who presented with excessive fatigue under treatment with valproate [5]. While their report noted that symptomatic hypothyroidism can be caused by valproate, whether this can be improved by discontinuation of valproate has remained unclear, because their report did not include a description about the treatment for hypothyroidism. Our case firstly demonstrates that valproate-induced hypothyroidism can be rapidly improved by discontinuation of valproate, even if it is symptomatic.

Clinical manifestations of hypothyroidism such as fatigue and psychomotor retardation are similar to those of depression. Therefore, hypothyroidism that develops during the course of treatment for depression is often overlooked. In such cases, patients can be considered as having treatment-resistant depression [6]. In patients with bipolar disorder who develop treatment-resistant depressive episodes, mood stabilizers such as valproate and lithium are continued, and patients are often supplemented with other treatments. Thyroid function is regularly screened for lithium, but is less likely to be tracked in patients using valproate [7]. Although the incidence of hypothyroidism due to valproate is speculated to be low, our case suggests that valproate-induced hypothyroidism should be considered when patients complain of excessive fatigue under treatment with valproate.

The mechanism of valproate-induced hypothyroidism is unclear. In our case, F-T4 was decreased, but TSH did not increase in reactivity, suggesting central hypothyroidism. Central hypothyroidism is caused by a malfunction of the hypothalamic–pituitary–thyroid (HPT) axis. Previous studies have suggested that γ-aminobutyric acid (GABA), whose action is enhanced by valproate, affects the HPT axis [8]. Given this finding, the central hypothyroidism in the present case might have been caused by the enhancing effect of valproate on the action of GABA. On the other hand, another mechanism in children using valproate has also been previously reported. Secondary deficiency of zinc and selenium due to valproate may decrease the synthesis of thyroid hormone and cause hypothyroidism with elevated TSH [9, 10]. However, in adults with bipolar disorder, the mechanism of valproate-induced hypothyroidism has been poorly studied. The effects of valproate on thyroid function have important implications for the treatment of bipolar disorder, so the elucidation of this mechanism is needed in future studies.

## Conclusion

We reported the second case of symptomatic valproate-induced hypothyroidism. Our case suggests that valproate-induced hypothyroidism can be rapidly improved by discontinuation of valproate, even if it is symptomatic. Valproate-induced hypothyroidism should be considered when patients complain of excessive fatigue under treatment with valproate.

## Data Availability

Not applicable.

## Abbreviations

F-T4: Free thyroxine
F-T3: Free triiodothyronine
TSH: Thyroid-stimulating hormone
HPT: Hypothalamic–pituitary–thyroid
GABA: γ-Aminobutyric acid
